# Common developmental trajectories and clinical identification of children with fetal alcohol spectrum disorders: A synthesis of the literature

**DOI:** 10.3389/adar.2023.10877

**Published:** 2023-04-03

**Authors:** Douglas Waite, Larry Burd

**Affiliations:** ^1^ Developmental Pediatrics, Bronxcare Health System, Mount Sinai School of Medicine, New York, NY, United States; ^2^ Department of Pediatrics, School of Medicine and Health Sciences, University of North Dakota, Grand Forks, ND, United States

**Keywords:** prenatal alcohol exposure, fetal alcohol syndrome, fetal alcohol spectrum disorders, neurodevelopmental disorder associated with prenatal alcohol exposure, prenatal substance exposure, foster care

## Abstract

At an estimated prevalence of up to five percent in the general population, fetal alcohol spectrum disorders (FASD) are the most common neurodevelopmental disorder, at least if not more prevalent than autism (2.3%). Despite this prevalence in the general population, pediatricians and other developmental specialists have thus far failed to diagnose this disability, leaving most children and adults without the supports provided for most other disabilities. This paper will provide a review of clinically relevant literature that describes the developmental challenges of children with fetal alcohol spectrum disorders and addresses similarities to and differences of FASD from other neurodevelopmental disorders such as autism and attention deficit hyperactivity disorder. A subsequent discussion will describe how a diagnosis of an FASD can establish a basis for understanding the developmental and behavioral challenges of children with an FASD, and how specific interventions can help support child development and maximize adult independence.

## Introduction: The prevalence of children with fetal alcohol spectrum disorders

In the 50 years since the effects of prenatal alcohol exposure upon fetal development were first described as a constellation of facial features, growth impairment, and neurodevelopmental impairments designated fetal alcohol syndrome (1), the effects of alcohol upon prenatal brain development and its subsequent neurodevelopmental sequelae have been expanded to include developmental challenges even in the absence of facial features and/or growth impairment associated with fetal alcohol syndrome. This broader category are the fetal alcohol spectrum disorders (FASD).

Fetal alcohol spectrum disorders are so common that a physician can be certain he or she has cared for a child with this disorder. Physicians can be just as certain that no professional previously diagnosed that child with an FASD (unless they were the person who suspected this diagnosis). Despite widespread warnings, women often receive conflicting messages from professionals on the safety of alcohol use during pregnancy and alcohol use during pregnancy continues to be prevalent. A recent CDC study found that 13.5% of pregnant adults reported current drinking and 5.2% reported binge drinking in the past 30 days (2). Estimates of alcohol consumption by non-pregnant women of child-bearing age (18–44 years) range from 53% of any alcohol use to 18.2% of women who binge drink with the majority of women being college-educated and employed (3). This statistic becomes especially important since many women do not discover their pregnancy until after missing their regular menstruation at 4–6 weeks gestational age, during which time brain development has already been effected by neurotoxic exposure. Common reasons for alcohol use in pregnancy are lack of awareness of the adverse effects of alcohol upon the fetus, the belief that only excessive alcohol use is harmful, maternal stress during pregnancy, unwanted or unplanned pregnancy, and alcohol dependence (4).

A study of first grade children in schools across four sites in the Midwest United States, found a total prevalence of FASD of 1.1%–5% (up to one in twenty children) (5). While the span in prevalence estimates likely reflects regional variation across the US in alcohol use during pregnancy, this figure highlights that FASD is a disorder as common as any other medical condition physicians diagnose and treat each day. The US Census Bureau estimates that in 2020, there were 72.8 million children living in the United States^1^, making the range of the number of children with FASD between 0.8 and 3.64 million children based upon FASD prevalence documented above. Yet compared to asthma, a disease with an estimated prevalence of 1 in 12 children (8.3%) (6) or the 1 in 44 children with autism (2.3%) (7), physicians and other professionals rarely consider FASD among their differential diagnosis as a cause of developmental and behavioral challenges.

FASD is even more prevalent among children in foster care, where an estimated 16.9% of children are affected by an FASD (8). Yet a diagnostic clinic evaluating children referred from foster care for developmental and behavioral challenges, found 80% of children who were subsequently diagnosed with an FASD, had never been previously identified with this disorder (9). Parental substance and alcohol use disorders are one of the most common reasons for foster care placement (10). Screening of all children entering child welfare for prenatal exposure to alcohol and other substances is far from routine. Even when prenatal exposure to other substances such as cannabis, cocaine, or opioids is documented in newborn medical records, screening for prenatal alcohol exposure is notably absent in most obstetric, pediatric, and child welfare records (11, 12).

Current clinical guidelines for diagnosis and assessment state that “assignment of an FASD diagnosis is a complex medical diagnostic process best accomplished through a structured multidisciplinary approach by a clinical team comprising members with varied but complementary experience, qualifications, and skills” (13). But multiple and often discrepant classification systems and standards of what constitutes a diagnosis of FASD in the United States create a major barrier to diagnosis because of the lack of uniform language to define FASD. Countries such as Canada and Australia have adopted uniform diagnostic criteria that allow a nation-wide, consistent approach to diagnosis with detailed clinical practice guidelines to support assessment, treatment, and disability qualification (14,15). Persistent recommendations for a comprehensive, multidisciplinary assessment might be appropriate if such resources were readily available in the United States. However, given the prevalence of FASD and the scarcity of diagnostic resources in the United States, how can children with an FASD be more readily identified and treated? Can children with an FASD be differentiated from children with other neurodevelopmental disorders? Is there sufficient consensus in the literature to suggest a diagnostic path that can allow physicians and other professionals a means to provisionally identify children with FASD in the absence of a multidisciplinary team and initiate treatment recommendations?

This article reviews common developmental trajectories of the neurodevelopmental disorders, including autism, global developmental delay, speech delay, ADHD, intellectual disability, and FASD. After reviewing similarities and differences across the neurodevelopmental disorders and a process for screening for prenatal alcohol exposure, the criteria of neurodevelopmental disorder associated with prenatal alcohol exposure is described as a pathway for practitioners to begin identifying and treating children with suspected FASD.

## Methodology

**FIGURE 1:**
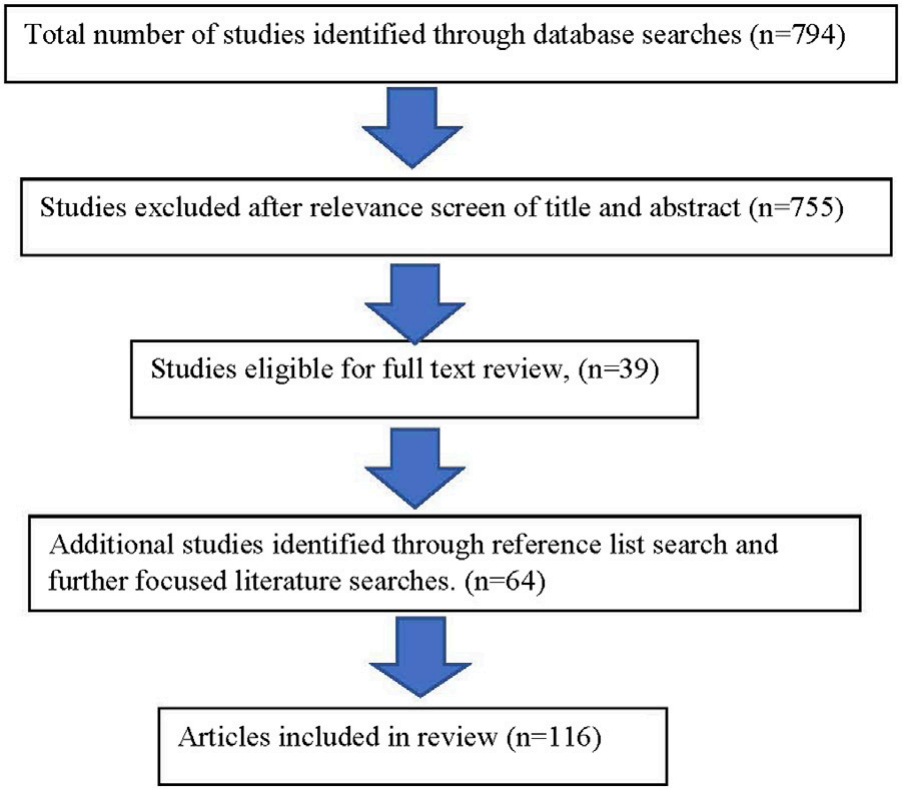
Depiction of methodological search of literature.

Literature searches were completed through PubMed for all articles utilizing the search terms of “prenatal alcohol exposure” crossed with “developmental trajectory” and “diagnosis” for the years 2010–2022 in all languages yielded a total of 21 results in PubMed. A more restricted search limited to review articles using the terms “neurodevelopmental disorders crossed with “developmental trajectory” and “diagnosis” for the years 2010–2022 in all languages yielded a total of 773 articles. A total number of 794 articles were subsequently reviewed by title and abstract for relevance to the topic of this article (Figure 1).

These results, including citations and references, were reviewed based upon their relevance to the similarities and differences of development among children with an FASD compared to other neurodevelopmental disorders. Additional focused searches were made based upon the need for supporting documentation during the writing of the article.

## Results

A structured clinical approach to diagnosis and intervention was synthesized based upon the diagnostic criteria for neurodevelopmental disorder associated with prenatal alcohol exposure (16,17) to help clarify a clinical means of identifying and initiating interventions for children with suspected FASD in general pediatric practice and during other developmental assessments.

### Common neurodevelopmental disorders and their developmental trajectories

**FIGURE 2:**
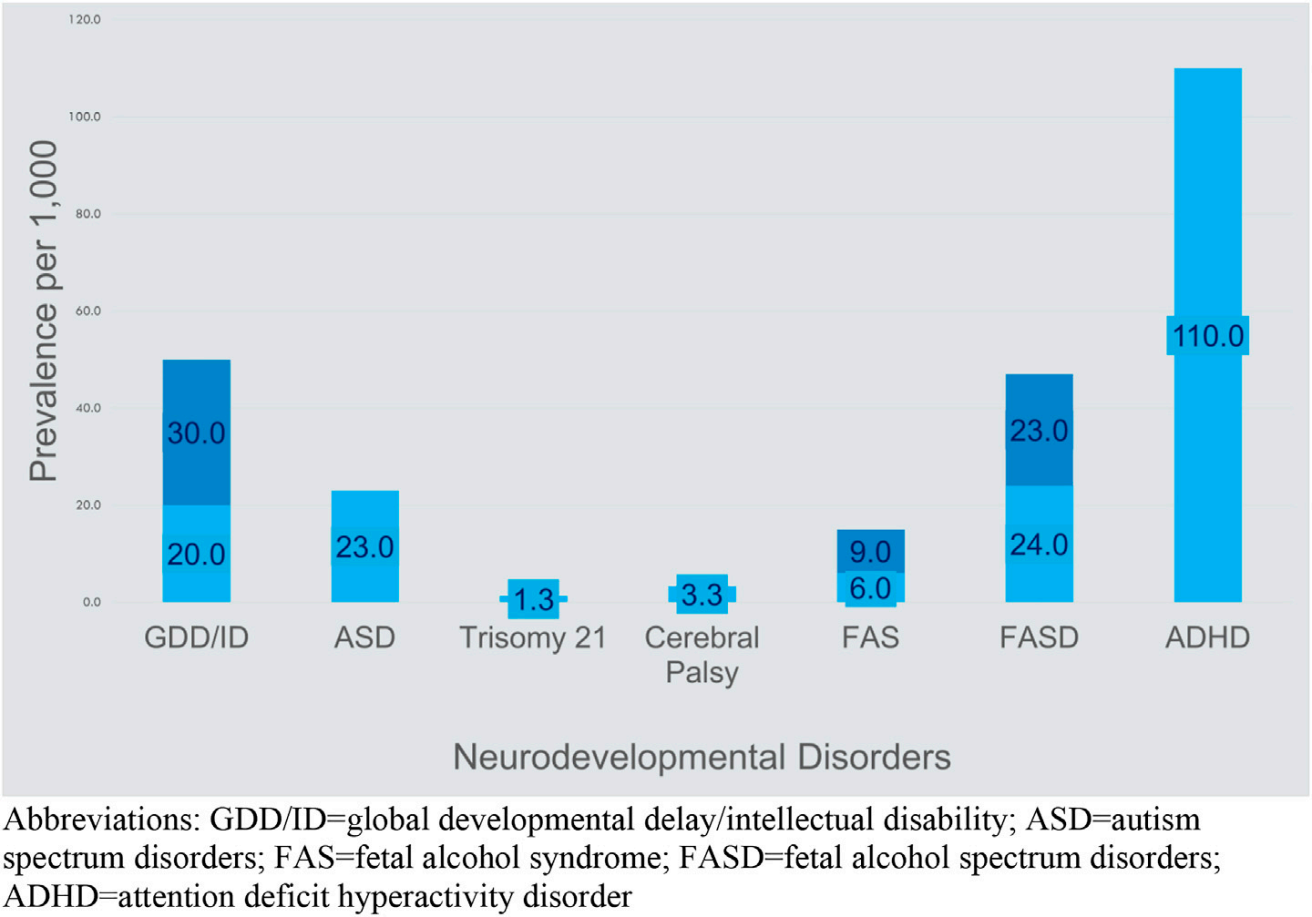
Prevalence of common causes of disability (21–25)^2^. Abbreviations: GDD/ID, global developmental delay/intellectual disability; ASD, autism spectrum disorders; FAS, fetal alcohol syndrome; FASD, fetal alcohol spectrum disorders; ADHD, attention deficit hyperactivity disorder.

The category of neurodevelopmental disorders spans developmental challenges that present during early childhood as manifestations of manifold and yet to be clearly defined differences in brain development. Estimated prevalence rates for the neurodevelopmental disorders range from 0.63% to 3% for intellectual disability, 5%–11% for ADHD, 3%–10% for specific learning disorders, 42% for communication disorders, and 0.76%–17% for motor disorders (18). Causes of neurodevelopmental disorders range from genetic abnormalities, pre- or post-natal infections, asphyxia, prematurity, epigenetic changes prior to or after birth, prenatal or post-natal exposure to neurotoxins, nutritional deficiencies, and prenatal fetal or maternal medical conditions including maternal mental health (19). The interplay across this list is complex and captured in the relatively new fields of epigenetics and the neuroendocrine immune system (20). The result is manifested as disabilities such as global developmental delay, intellectual disability, autism, attention deficit disorders, learning disabilities, speech/language disorders, and fetal alcohol spectrum disorders. Figure 2 shows the lower and upper estimates for the primary neurodevelopmental disorders discussed in this article compared to more commonly known specific disabilities of Trisomy 21 and cerebral palsy.

The pretense of these terms becomes apparent in clinical practice as these disorders manifest many common characteristics and often appear simultaneously with a great overlap of developmental challenges. The wide prevalence estimates given above for each neurodevelopmental disorder further hint at the great overlap among these diagnoses (Figure 2). This overlap leads inevitably to the question of whether the neurodevelopmental disorders are distinct disorders. Comorbidity among the neurodevelopmental disorders is the rule rather than the exception and the spectrum of these developmental challenges exists along a continuum of severity (26). In addition, developmental challenges often shift and become more differentiated over time leading to a clearer, more specific diagnosis with advancing chronological age. In clinical practice, the question becomes to what extent a specific diagnosis provides a context for a discussion of a child’s behavioral challenges with the family and a starting point to obtain intervention services (27).

The primary challenge in assessing a child with developmental delay becomes one of differentiating a child with autism, FASD, or global developmental delay, from a child with isolated speech delay (communication disorder). Diagnosis often relies upon the pattern of developmental challenges as measured across developmental domains on standardized tests that document function outside of the normal range of development (28). The lack of specific biological markers and lack of specific distinct etiologies makes developmental assessment more challenging and subjective despite the use of standardized developmental tests. Nevertheless, the concept of a neurodevelopmental disorder as developmental delays that present over the course of development is useful in seeking to identify children who require intervention, gives a language to communicate with parents and other professionals, and provides a pathway for obtaining community-based services. A detailed history that focuses upon the pattern of a child’s early developmental trajectories across domains of developments forms the basis for distinguishing the neurodevelopmental disorders to facilitate diagnosis and intervention. Prior to reviewing the spectrum of developmental delays found in the neurodevelopmental disorders, this article will briefly review the trajectory of normal development from birth to age 3 years.

Because the diagnosis criteria for ADHD which include impairments in attention and self-regulation (hyperactivity/impulsivity) are often present across most neurodevelopmental disorders, I will focus on this neurodevelopmental disorder last as a diagnosis of exclusion of the four core neurodevelopmental disorders: fetal alcohol spectrum disorders, autism, global developmental delay, and speech/language disorders.^2^

### A simplified clinical approach to developmental assessment

**FIGURE 3:**
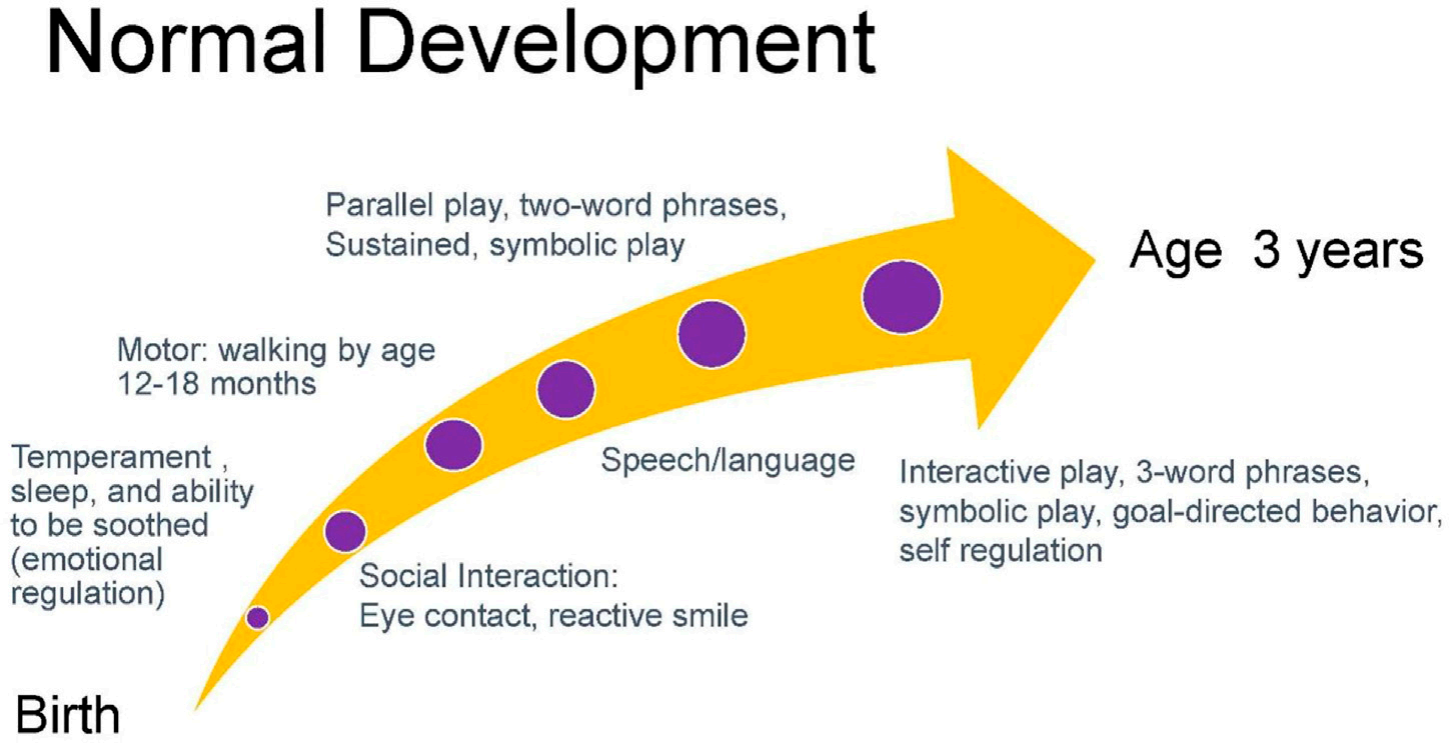
Milestones of normal development.

Gessell noted that normal development proceeds in an orderly, timed, and sequential process that occurs with such regularity that it is predictable (29). While there is variation from child to child within the framework of normal development, a common normal trajectory is depicted below in Figure 3 (30). The departure from expected developmental trajectories helps to identify children needing assessment. Current recommendations by the American Academy of Pediatrics recommend developmental screening of all children using a validated developmental screening test at the 9-, 18-, and 30-month visits. This recommendation aids in identification of children at risk of developmental delays and autism. Screens typically include the Ages to Stages Questionnaires and Modified Checklist for Autism (31).

Evaluation for speech delay is the most common cause of referral for developmental evaluation. The initial task in evaluating speech delay is determining whether this is a case of isolated speech delay (communication disorder), or a neurodevelopmental disorder that spans other domains of development such as autism, global developmental delay, and FASD.

**FIGURE 4:**
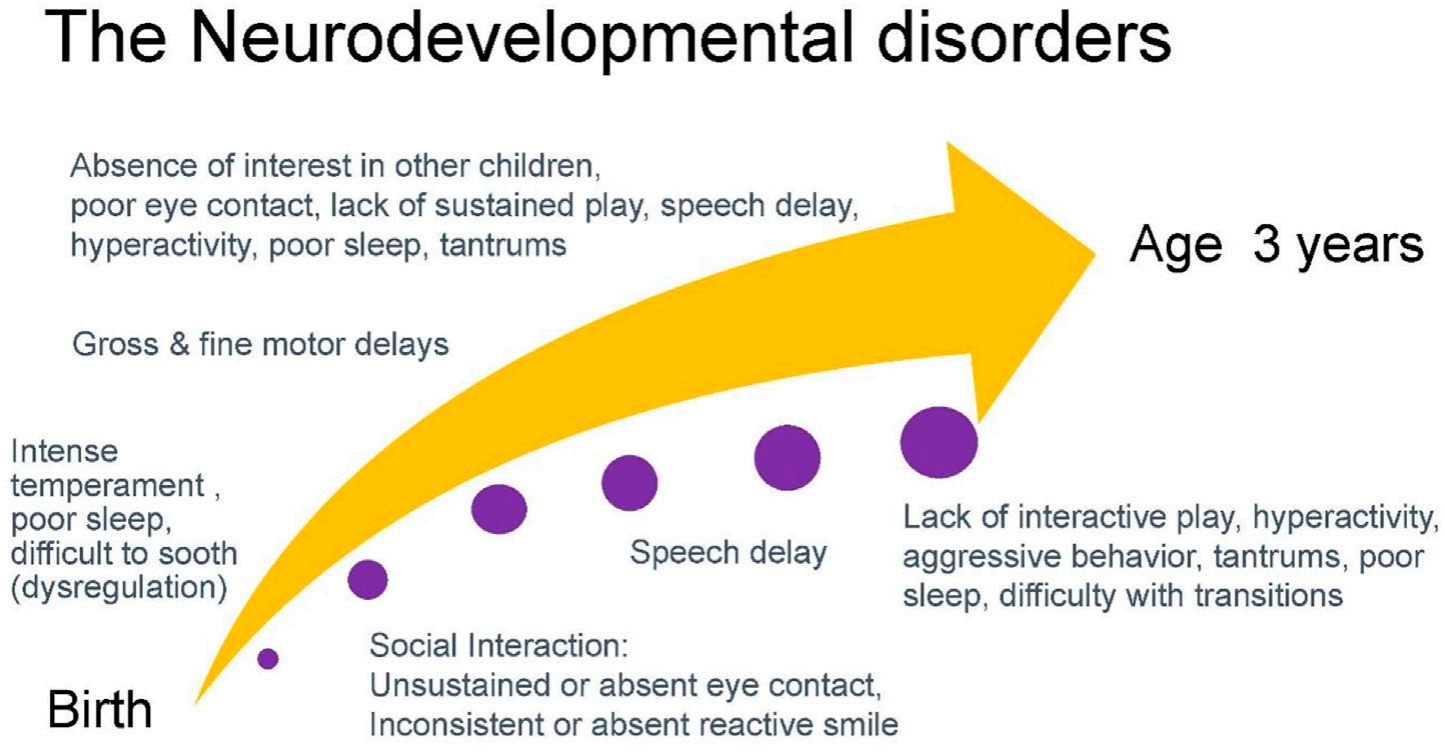
An example of a common developmental trajectory of a child with a neurodevelopmental disorder.

In obtaining a developmental history, initial queries can focus on the temperament of a child including patterns of sleep, ability to be soothed, and activity level. Infant temperament is associated with attachment which is the basis for gains in social interactions and subsequent acquisition of speech and language (32, 33). Absence of eye contact and reactive smile in the first months of infancy is often one of the first signs of autism (34, 35). Eye contact and social engagement lead to reactive cooing and the first stages of verbal interaction that subsequently progress to use of repetitive syllables (babbling) between 6 and 9 months and intelligible speech around age 1 year (36). Motor milestones proceed in a cephalo-caudal direction with progression from gaining head control by 3 months, to lifting chest when prone by 5 months, to sitting at 6 months, crawling at 9 months, and walking by 1 year. The development of speech and ambulation allows the infant to explore the world and engage more readily in interactions with others. Social development includes eye contact, use of non-verbal gestures such as indicative pointing, and motivation to seek out social interactions. These processes are concurrent with increased attention and cognitive ability that allow the acquisition of sustained and symbolic play by age 2 years. While children around age 2 years tend to engage in parallel play (minimally interactive play in proximity to other children), by age 3 years children engage in interactive play and goal-directed behavior (37). Concurrent with this development is the emerging ability to regulate emotions such as frustration that facilitates social interactions (38). Children with neurodevelopmental disorders frequently present with variable patterns of delays in development across developmental domains. Figure 4 charts an example of the developmental trajectory of a child with a neurodevelopmental disorder. This deviation from expected patterns of development allows practitioners to identify a constellation of developmental challenges that helps establish a differential diagnosis.

Children with global developmental delay present the clearest example of altered trajectories of development. Pervasive delays across two or more developmental domains of cognitive, adaptive, social-emotional, gross and fine motor, and speech domains characterize the challenges of children with global developmental delay (39). The diagnosis of global developmental delay is limited to children under the age of 5 years who are unable to undergo systematic assessments of intellectual functioning, including children who are too young to participate in standardized testing (16,40). While use of standardized testing measures such as those employed in evaluation by early intervention and schools can verify clinical suspicion, cognitive ability in children younger than 3 years is in flux and isolated testing is often unreliable (41). A common clinical practice is the use of the developmental quotient obtained by dividing the child’s estimated developmental age over their current chronological age. Developmental quotients below 70 strongly suggest delays in that specific domain. While intellectual disability may later be diagnosed in a child with global developmental delay (the prevalence of global developmental delay, like that of intellectual disability, is estimated to be 1% to 3%) (21), not all children with global delays will go on to meet criteria for an intellectual disability (42).

Overall prevalence rates for specific language impairment in kindergarten children is estimated to be 7.4% with higher prevalence for boys (8%), compared to girls (6%) (43). A study of 7,267 children aged 4–5 years found the estimated prevalence of language disorder of unknown origin to be 7.58% while the prevalence of language impairment associated with intellectual disability and/or other medical diagnosis was 2.34% (44). While children with language disorder may have greater symptoms of social, emotional, and behavioral challenges compared to peers and often have later challenges in school achievement, they lack the major delays in the other developmental domains of socio-emotional, adaptive, and cognitive ability and the repetitive behaviors and lack of social interest found among children with autism. Thus, in assessing a child with speech delay, concurrent delays in other domains of development can help differentiate the primary neurodevelopmental disorders (45). An audiologist should evaluate all children with speech delay to rule out conductive or sensorineural hearing loss.

In contrast to the global delays or isolated speech delays described above, a diagnosis of autism rests upon impairments in social and communication domains along with signs of restricted interests and repetitive behaviors (16). These include impairments in social-emotional interaction such as eye contact, lack of socially reactive smile or emotion, and lack of interest in initiating or responding to social interactions. Restricted, repetitive patterns of behavior, interests, or activities can be seen as repetitive motor movements, use of objects, or speech such as pacing, spinning, repetitive hand-eye movements, and echolalia. Insistence on sameness and rigid routines leads to severe emotional dysregulation, and highly restricted, fixated interests that are abnormal in intensity or focus (strong attachment to single objects), as well as hyper- or hypo-reactivity to sensory input (sounds, textures, smell, touch, visual fixation on details or lights or movement) (46).

It is therefore unsurprising that up to 62.3% of children with global developmental delay also meet diagnostic criteria for autism (47). In some cases regression of development occurs after the first year of life, prior to which socio-communicative skills might have appeared normal to parents (48). Beyond neurodegenerative disorders such as Rett syndrome, few other neurodevelopmental disorders present with the regressive loss of communication or social interaction described by parents of children with autism. Broad estimates across studies suggest 11%–65% of school-age children with autism subsequently also have the additional diagnosis of intellectual disability (49). Children with autism or global developmental delay often have severe behavioral challenges, the severity of which inversely correlates with the child’s developmental quotient and cognitive ability (50). Symptoms of inattention and hyperactivity can easily be diagnosed as ADHD without recognition of an underlying diagnosis of autism or intellectual disability. Genetic evaluation and testing should be considered in all children with suspected autism, global developmental delay, and intellectual disability as well as children with suspected FASD to help exclude genetic causes of developmental challenges which can be present in addition to prenatal alcohol exposure.

### Neurodevelopmental disorder associated with prenatal alcohol exposure

#### A path to increase identification of children with FASD

**FIGURE 5:**
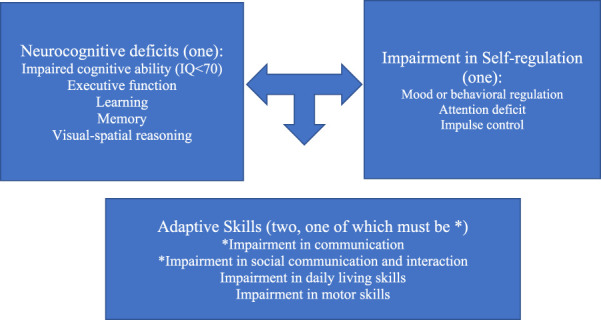
Criteria for neurodevelopmental disorder associated with prenatal alcohol exposure.

Given the breadth and overlap of the three primary neurodevelopmental disorders highlighted above, how can pediatricians begin to discern children with an FASD from other neurodevelopmental disorders? How can practitioners identify and establish interventions for children living with an FASD in the absence of an FASD multidisciplinary diagnostic center? The DSM-5 diagnostic criteria for neurodevelopmental disorder associated with prenatal alcohol exposure (ND-PAE) provides a straightforward path for practitioners to establish a provisional diagnosis much as pediatricians currently identify children with suspected autism (see Figure 5). One or more impairments in neurocognitive function, one or more impairments in self-regulation, and two or more deficits in adaptive function (with at least one being one of the first two symptoms highlighted by an asterisk) are sufficient to establish a diagnosis of ND-PAE if there is confirmed history of prenatal alcohol exposure (51).

Since its inclusion in the DSM-5 as a “condition for further study,” there appears to be strong correlation between the diagnostic categories of FASD and ND-PAE (52, 53). The advantage of ND-PAE criteria however is the emphasis on neurodevelopmental manifestations that practitioners see daily without extensive focus upon facial features and growth impairment that are often a barrier to diagnosis. While the clinical diagnostic guidelines for a diagnosis of ND-PAE may be less sensitive and specific than the traditional standards for the diagnosis of FASD, the utility of ND-PAE guidelines for front-line practitioners make this a step toward identifying children with an FASD (54).

A diagnosis of ND-PAE requires:

(1) One or more neurocognitive deficits
(2) One or more impairments in self-regulation
(3) Two or more impairments in adaptive skills, one of which must be communication deficit or impairment in social communication and interaction
(4) Documentation of more than minimal prenatal alcohol exposure

Children with FASD range in presentation from global developmental delay, symptoms of autism, isolated speech delay, or isolated early behavioral challenges similar to those seen among children with ADHD (Table 1) (55). Despite the great overlap across the neurodevelopmental disorders, differences in developmental challenges become more apparent with increasing chronological age. For example, young children with FASD may be diagnosed with autism because of speech delay, difficulty with transitions, sensory processing issues, socially inappropriate behaviors, difficulties with interpersonal interactions, and emotional/behavioral dysregulation. Challenges in social cognition are a primary challenge among children with FASD. Individuals with FASD have greater difficulties interpreting facial emotions than typically developing children (56). But while children with FASD share challenges in social skills and behavioral issues that can lead to a diagnosis of autism, children with FASD often score low in repetitive behaviors and restricted interests (57). Table 1 highlights common developmental challenges of children with FASD between ages 4–12 years.

**TABLE 1:** Middle school developmental challenges of children with FASD.

Developmental challenges of children with FASD: Ages 4–12 years
• Difficulties with receptive language compared to expressive language (auditory processing); Difficulties with conversation
• Difficulties in peer interactions (reading non-verbal cues, auditory processing, emotional/behavioral dysregulation, inappropriate interpersonal boundaries)
• Hyperactive, poor attention, disorganized (often referred for ADHD evaluation by age 3–4 years)
• Impulsivity, lack of awareness of danger and consequences
• Learning challenges (learns it then forgets it)
• Difficulties with tasks of daily living (“you should be able to do this at your age”)
• Confabulation
• Aggressive behavior
• High risk for school suspension or expulsion (as early as kindergarten)
• Sleep difficulties

Yet more subtle differences can distinguish FASD from autism. The difficulties in initiating social interaction, sharing affect, and using non-verbal communication common in children with autism are less common in children with FASD who tend to seek out social interaction at the exclusion of awareness of interpersonal boundaries. Similarly, while children with ASD are often referred to as aloof or uninterested in social interaction, children with FASD are more likely to make sustained eye contact, use indicative pointing to show or express interest or direct attention (theory of mind), engage in social interaction (often with an overly social and indiscriminately friendly presence), engage in interactive play and simple conversation, and offer comfort to others (58). Children with FASD are often overly friendly and lack interpersonal boundaries (as opposed to preference for solitary play). They are also at higher risk for behavioral challenges with symptoms of hyperactivity, impulsivity, and aggression easily mistaken for self-willed behaviors and attributed to a psychiatric disorder (ADHD) or an “emotional disturbance” instead of as a manifestation of neurological impairment. These challenging behaviors are the result of impairments in brain function across memory, learning, cognitive flexibility, comprehension, attention, planning, social skill development, and learning (59, 60). School evaluations often view learning challenges among children with an FASD as simple issues with attention and motivation, rather than as an underlying static encephalopathy or learning disorder. Because most children with an FASD are frequently not diagnosed with this disorder, their behaviors place them at higher risk for school suspension or school failure.

As noted at the beginning of this article, the prevalence of ADHD ranges from 5%–11% in the general population. ADHD is a neurodevelopmental disorder defined by impaired levels of inattention, disorganization, and/or hyperactivity-impulsivity. These are manifested by inability to stay on task, seeming not to listen, losing materials, being overly active, inability to stay seated or wait, and intruding into other people’s activities at levels that are excessive and inconsistent with age or developmental level (16). ADHD is commonly diagnosed among children with autism, global developmental delay, intellectual disability, and fetal alcohol spectrum disorder. At least 41%–48% of children with prenatal alcohol exposure are diagnosed with ADHD (61,62). Clinical experience suggests this percentage is far higher but limited by lack of screening for prenatal alcohol exposure in ADHD prevalence studies (63).

Children with FASD and ADHD both have challenges in executive function, including working memory, attention, behavioral regulation, and impulse control. Early challenges in executive function (the ability to focus attention, engage in sustained play, have goal-oriented behavior, and regulate emotions across different environments) and social function (the ability to engage in joint attention, exhibit social reciprocity and sharing, and perspective taking) are common to preschool children with FASD and become more apparent with age (64). Marked behavioral problems often eclipse the neurological impairments of FASD, commonly leading to multiple psychiatric diagnoses (ADHD, ODD, conduct disorder being the most common). Thus, behavioral referral of children with undiagnosed FASD nearly always leads to a diagnosis of ADHD in early childhood. The more recent concept of complex ADHD describes children with early onset of ADHD before age 4 years and have moderate to severe functional impairment, or inadequate response to treatment (65). Worsening of behavior or failure to respond to stimulant medication or lack of response to typical behavioral interventions should suggest a possible underlying diagnosis of FASD among children with a diagnosis of ADHD (66). While research is needed to clarify the extent of prenatal alcohol or other substance exposure among children with complex ADHD, the severe early developmental trajectory is strongly similar to those seen with FASD.

FASD is the most common identifiable cause of secondary morbidities such as intellectual disability, ADHD, anxiety disorders, and learning disabilities (63, 67). Children with multiple psychiatric diagnoses such as ADHD, oppositional deficient disorder, disruptive mood dysregulation disorder, conduct disorder, or intellectual disability should be screened for prenatal alcohol exposure to ensure a diagnosis of FASD is not the underlying cause of the severe emotional/behavioral dysregulation that attends each of these disorders (67). Furthermore, challenges in shifting activities and difficulties with transitions are common among children with autism and often a primary behavioral challenge of children with FASD, resulting in emotional/behavioral dysregulation during transitions or limit-setting (60). Children with FASD typically require a highly structured environment and become more dysregulated in overstimulating environments (68). The poor ability of many children with FASD to regulate sensory stimulation mirrors the challenges of children with autism who easily become dysregulated by sensory overstimulation. This may help explain why children with FASD often fail to respond well to stimulant medication but are more responsive to medication that targets emotional/behavioral dysregulation and symptoms of anxiety (69)^3^.

**FIGURE 6:**
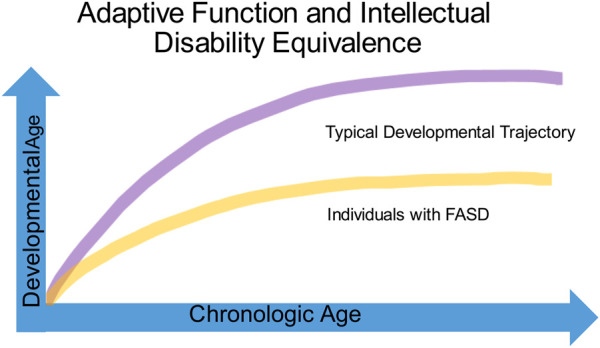
Adaptive function and intellectual disability equivalence.

Identifying impairments in adaptive function is critical to understanding the developmental challenges of children with FASD (Figure 6). Adaptive function is the ability to complete day-to-day age-appropriate tasks and spans skills such as receptive and expressive communication, social interaction, coping skills, and activities of daily living. While children with FASD, autism, intellectual disability and ADHD often show adaptive behavior impairments, the relationship of cognitive ability to adaptive function often helps distinguish children with FASD from the other neurodevelopmental disorders. The DSM-V defines intellectual disability as impairments in both cognitive and adaptive function. In contrast, children with autism without intellectual disability typically score low in the communication and socialization domains of adaptive function and often present with a significant gap between high non-verbal and low verbal abilities on cognitive testing (70). Estimates of cognitive ability among children with FASD vary widely, ranging from 20 to 120, with an average of about 72. Children with FASD typically have significantly lower IQ scores than those with ADHD and score lower in adaptive functioning when compared to IQ matched children (71). While adaptive skills improve with age for children with ADHD, children with FASD tend to be significantly more impaired in the daily living skills domain and impairments in both socialization and communication domains become more apparent with age (Figure 6) (72, 73).

Individuals with FASD often have normal to borderline cognitive ability (above 70) and frequently fail to meet criteria for intellectual disability or autism leading to assumptions that they can complete tasks “if only they try harder.” Coles et al. have described two subsets of children with alcohol-related neurodevelopmental disorder: one with cognitive impairment, the other with primarily behavioral manifestations. Of note, both children with primarily behavioral manifestations and those with cognitive impairments both scored low on adaptive function (74). The Collaborative Initiative on Fetal Alcohol Spectrum Disorders has proposed a decision tree to help identify children with PAE, but these criteria rely on psychometric measures such as the Child Behavioral Checklist and the Vineland Adaptive Behavioral Scale that are often unavailable to practitioners without access to neuropsychological testing (75).

Evaluations by the school can fail to identify impaired adaptive function in the face of normal to low cognitive ability. This “intellectual disability equivalence” often leads to difficulties in learning, social interactions, and behavior that become increasingly apparent as children move through adolescence (76) (see Figure 6). The inability to qualify for services available to individuals with autism and intellectual disability leaves a gap in services that is one of the greatest struggles for families caring for children with an FASD. The failure to identify challenges in adaptive function and the subsequent blame placed upon children from early childhood to adulthood for difficulties with basic age-appropriate tasks not only damages self-esteem, but over time leads to anxiety, depression, and even suicide in individuals living with an FASD.

Barriers to FASD diagnosis include lack of awareness of FASD prevalence, manifestations, and diagnostic criteria and discomfort of professionals in discussing prenatal exposures. In addition, there is a lack of systematic screening for prenatal alcohol exposure by obstetricians, pediatricians, psychiatrists, psychologists, and social workers; lack of a biological marker for diagnosis of FASD; and underreporting of alcohol use during pregnancy due to stigma and fear of repercussions (77). Early recognition of exposure allows risk stratification to identify children who need closer developmental follow up. Stigma against women with substance and alcohol use disorders continues to be a barrier to diagnosis, especially in the child welfare system where parents feel judged and threatened with termination of their parental rights. Each of these barriers leads to a lack of resources for interventions services specific to an FASD diagnosis. The sum of these failures leads to a need for constant advocacy by providers and families in gaining services for children whose neurological impairments frequently manifest as behavioral and psychiatric challenges.

### Screening for prenatal alcohol

When evaluating a child with developmental or behavioral challenges, screening for prenatal alcohol exposure (PAE) is the single most important first step in considering a diagnosis of FASD. Prenatal alcohol exposure alone can be a predictor of child development. A study comparing documented prenatal exposure using the biological marker, meconium ethyl glucuronide, and cognitive deficits and symptoms of ADHD, found a partially dose-dependent relationship to development (78). Therefore, a brief review of a process for obtaining a history of prenatal exposure deserves discussion.

Evaluation can easily incorporate screening as part of obtaining the prenatal and birth history that is routine for most practitioners. The effectiveness of weaving questions for PAE into the prenatal history makes asking questions that are often uncomfortable for both professionals and parents, easier to present as a routine part of information gathering. Below is a simple script practitioners can complete quickly in even the busiest of practices.

• How far into your pregnancy did you discover you were pregnant?
• Did you have any medical problems during your pregnancy?
• Were you prescribed any medications during your pregnancy?
• How much alcohol did you use prior to finding out you were pregnant?
• How much alcohol did you use after finding out you were pregnant?
• What other substances did you use before and after you found out you were pregnant (such as cannabis, opioids, or other non-prescribed medications)?

Note the importance of obtaining a history of alcohol and other substance exposure prior to pregnancy recognition. After obtaining a positive history of alcohol use prior to or after pregnancy recognition, further investigation of alcohol preference (beer, wine, liquor) and the size of a typical drink helps clarify the extent of alcohol-related neurotoxic exposure. Suggested guidelines for significant alcohol exposure have been made (79), but current evidence documents that even small amounts of prenatal alcohol exposure can affect brain development and that there is no known “safe” amount of alcohol use in pregnancy (80). While there is no safe amount of alcohol consumption during pregnancy, binge drinking with sharp elevated maternal blood alcohol levels readily cross the placenta and carry the highest risk for a fetal alcohol spectrum disorder. Greater maternal blood alcohol levels are associated with greater severity within the spectrum of FASD, with higher levels associated with fetal alcohol syndrome (81). In addition to a direct maternal interview, information of alcohol consumption during pregnancy can also be obtained from a reliable collateral source such as a family member, social service agency, or prenatal or maternal medical records. Definitions of significant alcohol exposure vary, but consumption of six or more drinks per week for more than 2 weeks or three or more drinks on two or more occasions can be considered a guideline for determining significant alcohol consumption (82). Additional history of significant alcohol consumption can also be obtained from documentation of social or legal problems associated with alcohol use or intoxication during pregnancy (83).

Screening for PAE is often an iterative process that may require revisitation and in which a parent who may initially deny use of alcohol during pregnancy, later discloses use in the context of a relationship of trust that focuses upon the wellbeing of the child. Even with documented prenatal alcohol exposure, a diagnosis of an FASD is inappropriate until further psychological standardized testing (including testing for adaptive function) and evaluations by early intervention or the school can be completed. The discussion of a diagnosis of FASD with a parent requires patience, frequently starting with interventions before clarifying the suspected etiology of developmental delays. Anticipatory guidance of potential developmental and behavioral challenges and the possible need for additional support in the future builds a working relationship with parents and diminishes the helplessness, guilt, and feeling of aloneness that comes with caring for a child with severe developmental and behavioral difficulties.

### Interventions: Bending the trajectory

The primary reason for any diagnosis is intervention. Diagnosis allows education of caregivers about the disabilities and anticipatory guidance for risks and current or future need for interventions. Diagnosis also allows individuals with FASD to better understand their strengths and weaknesses (“blind spots”). Ideally diagnosis also allows access to disability services. In most cases the subjectivity of a diagnosis rests upon clinical experience and awareness of the importance of diagnosis in obtaining services. Diagnosis also allows a common language for clinicians to discuss developmental challenges in the context of a diagnosis, including framing a prognosis, and providing anticipatory guidance for possible future challenges. Table 2 highlights common developmental challenges of children as they move through adolescence.

**TABLE 2:** Developmental challenges of adolescents and young adults with FASD.

Developmental challenges of adolescents with an FASD: Ages 13–21 years
• Increasing gap between chronological age and developmental age, especially in adaptive function (tasks of daily living, maintaining safety, difficulties with managing time and money), “18 going on 10”
• Difficulties with respective language (auditory processing), reading non-verbal social cues engaging in back-and-forth conversation
• Difficulties making and keeping friends
• Poor interpersonal boundaries, sexually inappropriate behavior
• Gullibility, easily swayed by others to do acts they would not do alone
• Confabulation, taking possessions of others, stealing
• High risk for school failure/drop out
• Parent-child relationship difficulties including increasing use of aggression and destructive behavior in the home
• Emotional/behavioral dysregulation
• Increased risk of alcohol and/or substance use

**FIGURE 7:**
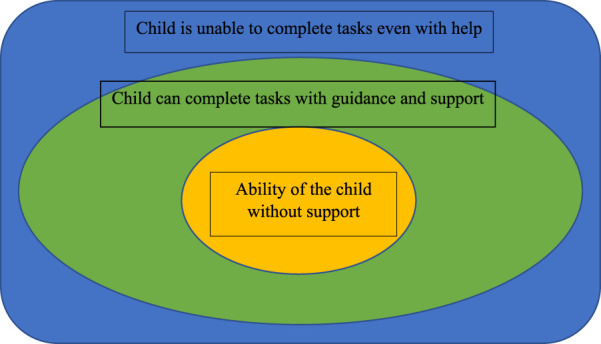
Conceptualizing the zone of proximal development.

Vygotsky’s model of the zone of proximal developmental provides a framework for helping parents and teachers greater awareness of developmental challenges and providing services that meet the child at the level of their developmental ability (Figure 7) (84–86). The zone of proximal development is based upon the three zones of the ability of the child to complete tasks without help or guidance, with support and guidance, and the level beyond which the child is unable to complete a task even with adult support. Identifying these impairments helps guide interventions that meet a child’s level of function and is imperative in working with all children with disabilities.

**FIGURE 8:**
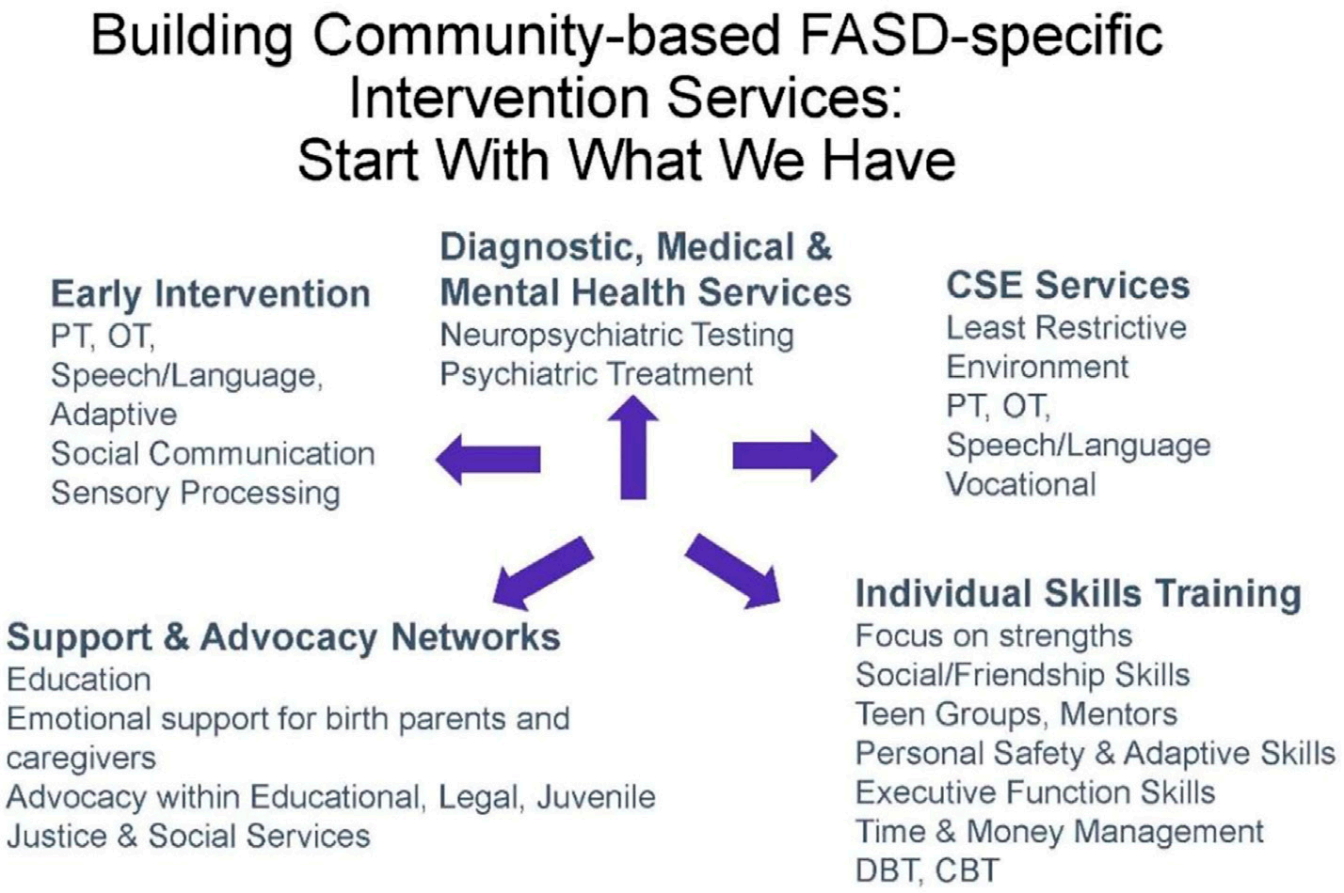
Building community supports.

While children with neurodevelopmental disorders such as speech delay, autism, and intellectual disability have a clear pathway to services, families of children with an FASD often find providers who lack training in caring for children with an FASD. There is an urgent need to establish a community network of service providers familiar with the challenges and interventions to support the development of children with an FASD (Figure 8). This network of services would ideally begin with early identification of children with an FASD prior to age 3 and include early intervention providers familiar with the challenges that attend a diagnosis of FASD. It would include family support services across childhood while ensuring transition from early intervention services to special education services and beyond (87). A major barrier to supportive services for families of a child with an FASD is the lack of inclusion of FASD as a developmental disability eligible for state-based disability services under the Individuals with Disabilities Act. While many states allow eligibility for disability services for children and adults with fetal alcohol syndrome, children with an FASD other than fetal alcohol syndrome often do not qualify for services unless they meet criteria for autism or intellectual disability despite documentation of severe impairments in adaptive function and life skills necessary to transition to adult independence.

Perhaps just as important as addressing current developmental needs of a child or adolescent with an FASD, providers should anticipate future challenges as adaptive function falls further behind age-expected abilities. Medications to target symptoms of ADHD, mood dysregulation, anxiety, depression, and sleep issues are common adjuncts to the greater implementation of environmental supports. Essential environmental supports include a calm highly structured environment with consistent routines at both home and at school. Providers often serve as advocates for services beyond school mandates for a least restrictive environment. Interventions should also address educational and vocational needs by highlighting adaptive function disabilities that often exist in the presence of borderline to normal cognitive abilities. Multiple interventions have been documented to specifically address the challenges of children with an FASD while supporting their families (88). Interventions should also address educational and vocational needs.

The transition from adolescence to adulthood is a time fraught with risks for school failure, anti-social or criminal behavior, substance use, victimization, worsening psychiatric illness, unemployment, and homelessness. Anticipation of each of these difficulties allows open discussion with parents who are frequently hesitant to discuss their concerns. Even following diagnosis of FASD, adolescents and adults with FASD remain at extreme risk for adverse outcomes. This risk is compounded by exposure to adverse childhood experiences, especially when these are frequent and enduring. Children with FASD are estimated to be at least 3.7 times more likely to have adverse childhood experiences than children without an FASD (89). Adverse experiences compound the developmental challenges of FASD to synergistically increase risk for developmental and behavioral challenges (90). Anticipatory guidance and ongoing work with families should include vigilance for adverse outcomes including school failure, recurrent school suspensions or expulsions, involvement in the juvenile justice system, sexual and interpersonal victimization, homelessness, and drug and alcohol use disorders (91,92).

In addition to poor family support, adaptive difficulties in completing age-expected tasks turn simple tasks (e.g., getting to work or appointments on time, interacting appropriately with others) into insurmountable obstacles without appropriate services. Although adolescents with FASD may appear confident about managing age-appropriate tasks, this apparent self-confidence often masks impairments in adaptive skills and low self-esteem (93). Therefore, transition plans should include vocational assessment and life skills training (e.g., how to schedule and ensure appointment punctuality, following directions, appropriate workplace social behavior) as well as support in finding employment and job coaching (94). Adaptive assessments during the diagnostic evaluation process should contain information about specific weaknesses in daily living skills (e.g., hygiene, nutrition, shopping, cooking, paying bills) that are especially important during transition. Most adolescents with FASD will need ongoing support and supervision for adaptive tasks that overwhelm their capability as they attempt to meet adult responsibilities (95). Without ongoing support during transition to adulthood to accommodate deficient executive functioning and associated adaptive impairments, treatment services—no matter how extensive—are unlikely to result in a successful transition to independence and productive integration into society. Independent living programs, subsidized rent programs, and home healthcare services can facilitate a successful transition to adulthood and maximize independence (96).

## Conclusion

General pediatricians and early childcare workers are often the first persons to assess a child with developmental delays. This means they are the gatekeepers for assessment and intervention long before specialists evaluate children. The lack of availability of specialists to diagnose FASD and greater lack of multidisciplinary FASD diagnostic centers, makes identification of children with an FASD in the general population an urgent public health concern. Just as interventions in other neurodevelopmental disorders improve outcomes, early identification and intervention is imperative to supporting children with an FASD. Practitioners can easily screen all children for prenatal alcohol exposure. Similarly, practitioners can begin to diagnose children with developmental delays who have a history of prenatal alcohol exposure and meet the criteria for ND-PAE. While many might argue that a diagnosis of FASD or ND-PAE is less helpful in obtaining services compared to other diagnoses such as autism, the developmental challenges and developmental trajectory of children with FASD are different from those with other neurodevelopmental disorders. Others might argue that the criteria for ND-PAE lack the sensitivity and specificity of traditional FASD diagnostic criteria. Yet traditional requirements for evaluation by a multidisciplinary diagnostic team have failed to identify the majority of children with an FASD and such centers as they currently exist will never be able to meet the demand for diagnostic and intervention services. A diagnosis of FASD offers a structure for discussion of developmental challenges with the family within the context of seeking interventions to maximize independence and prevent secondary morbidities. After 50 years of research that has increased our understanding of the effects of prenatal alcohol exposure upon neurodevelopment, front-line practitioners can use the knowledge gained from research to address the developmental and behavioral challenges of families that come to them seeking help for their children.

## Data Availability

The original contributions presented in the study are included in the article/supplementary material, further inquiries can be directed to the corresponding author.

## Footnotes

1
https://www.childstats.gov/americaschildren/tables/pop1.asp (Accessed 26 July, 2022).

2
https://www.cdc.gov/ncbddd/adhd/data.html (Accessed 15 January, 2023).

3
https://canfasd.ca/algorithm/ (Accessed 16 January, 2023).

## Abbreviations

ADHD, attention deficit-hyperactivity disorder; ASD, autism spectrum disorder; FASD, fetal alcohol spectrum disorders; FAS, fetal alcohol syndrome; ND-PAE, neurodevelopmental disorder associated with prenatal alcohol exposure; ODD, oppositional defiant disorder; PAE, prenatal alcohol exposure.
